# Changes in nutritional composition, functional, and sensory properties of yam flour as a result of presoaking

**DOI:** 10.1002/fsn3.150

**Published:** 2014-07-30

**Authors:** Adewale Olusegun Obadina, Bukunola Olaide Babatunde, Ifeoluwa Olotu

**Affiliations:** Department of Food Science and Technology, Federal University of AgricultureAbeokuta, Nigeria

**Keywords:** *D. alata*, *D. rotundata*, nutritional composition, paste, soaking, yam flour

## Abstract

This study investigated the effect of soaking pretreatments on some of the properties of flour obtained from two varieties of yam namely;*Dioscorea alata* and*Dioscorea rotundata* with a view of providing information that will enhance their end use. The yam varieties were washed, chipped, parboiled at 50°C, soaked for different periods (0, 6, 12, and 18 h), dried at 60°C, and milled into flour. The flour samples were analyzed for their nutritional composition, pH, color, and functional properties. The flour samples were also made into pastes and were sensorially analyzed and 0 h soaked samples were used as control. The protein content of 18 h-soaked*D. rotundata* and*D. alata* flour samples was significantly different from the control and soaking had no effect (*P* > 0.05) on the fat and ash content but the carbohydrate content of the flour samples ranged from 83.08% to 86.13%. The 18 h-soaked*D. rotundata* flour sample had the lowest peak viscosity, breakdown value, and final viscosity among the*D. rotundata* variety samples. Pasting temperature ranged from 79.80 to 83.60°C and 6-h soaked*D. alata* flour sample had the lowest water absorption capacity and the highest bulk density. On the basis of sensory analysis, the panelist preferred the taste, texture, color, and appearance of paste made from the 18-h soaked*D. rotundata* flour to the paste of other flour samples. The results of this study show that D.*rotundata* should be soaked for 18 h prior to drying and milling in order to obtain a good-quality flour and paste.

## Introduction

The genus*Dioscorea* (family*Dioscoreaceae*) consists of some species that are commonly known as yam which are consumed perennial in Africa, Asia, Latin America, and Oceania (http://en.wikipedia.org/wiki/Yam_%28vegetable%29). Throughout the world, over 150 species are grown (Purseglove 1991) and about six species are known as important staples in the tropics.*D. rotundata* (white yam),*D. esculenta* (Chinese yam),*D. alata* (water yam),*D. bulbifera* (aerial yam), and*D. dumenterum* (trifoliate yam) are among the economically important species (Ike and Inoni 2006). Yam serves as an important source of carbohydrate and serves as a major source of income in countries where they are cultivated. In 2007, 96% of the worldwide production of yam (52 million tons) was from Africa while 94% of the yam was from West Africa with Nigeria alone producing 71% (http://www.iita.org/yam). Yams are usually processed into dry-yam tubers/slices and flour in West African countries such as Ghana, Benin, and Nigeria (Bricas et al. 1997). Yam tubers are usually processed into flour called “gbodo” in Yoruba land of Nigeria by peeling, slicing, parboiling in hot water (40–60°C for 1–3 h), soaking, and sun drying (Onayemi and Potter 1974).

Majority of foods sold in the market presently are exposed to a certain degree of processing (Akingbala et al. 1995) and processing is also prerequisite for yam consumption. Gbodo—traditionally processed dry yam—gives an intermediate flour product upon milling which is called “elubo.” Elubo is usually stirred in boiling water to obtain a paste which is usually eaten with soups called “amala” (Akissoe et al. 2000). The quality attributes that consumers look out for in these products are their color, texture, and taste (Akissoe et al. 2000). Yam is still being processed to gbodo and elubo through traditional methods and their quality attributes differ from one processor/location to another (Hounhouigan et al. 2003). However, to the best knowledge, little information is available on how soaking as a processing variable affects the quality attributes of yam flour and this type of knowledge becomes important when the development of flour is considered. This study investigated the effect of different soaking time on the chemical composition, functional, and sensory properties of flour from*D. rotundata* and*D. alata*.

## Materials and Methods

Yam was obtained from Kuto market in Abeokuta, Ogun State, Nigeria. The two varieties of yam (*D. alata* and*D. rotundata*) were converted to chips using the method described by Ige and Akintunde (1981). The white and water yam tubers were washed with portable water. The yam was cut into chips (150 g) and parboiled at a temperature of 50°C. The parboiled samples were soaked in portable water at different times (0, 6, 12, and 18 h) 0 h-soaked samples of the two yam varieties were used as control. The soaked yam slices were dried to a constant weight using a cabinet drier (Jinan Food Machinery Co., Ltd., Jinan, China) at 60°C for 2 h. The dried yam chips were milled into flour, sieved using 0.25 *μ*m sieve, and were subjected to analyses.

### Nutritional composition analysis

The nutritional composition of yam flour, including protein and fat, fiber, ash, and carbohydrate was determined according to the methods of AOAC (2000).

### Functional properties analysis

#### Bulk density

The bulk density of the yam flour was determined with the method of Wang and Kinsella (1976).

#### Water absorption capacity

The water absorption capacities of the flour samples were carried out by the modified method of Prinyawiwatkul et al. (1997).

#### Dispersibility

A method described by Kulkarni and Ingle (1991) was used to measure dispersibility.

#### Pasting property

The pasting properties of the samples were measured using a Rapid Visco Analyzer, RVA (Model RVA-SUPER3; Newport Scientific 1998, Australia) of the yam flour.

#### pH

The pH of the sample was measured with a pH meter.

#### Color intensity

One gram of each sample was weighed into a 100 ml beaker. 25 ml Hcl was measured and added to the beaker to extract the color by shaking and homogenizing with glass rod for 30 min. The mixture was allowed to stand for 10 min after which it was filtered through hardened Whatman No 42 filter paper into another 100 ml conical flask. The organic filtrate obtained was used to determine color by taking the absorbance at wavelength of 520 nm on a spectrophotometer (Cecil 2483, Cambridge, UK).

### Sensory analysis

The yam flour samples were prepared by stirring them in hot water to make amala paste and served to 20 taste panelists who are regular consumers of amala. The pastes were rated for aroma, texture, color, and taste using a 9-point hedonic scale according to Iwe (2002).

### Statistical analysis

All analyses were carried out in triplicates and the data were subjected to analysis of variance (ANOVA). SAS version 9.0 (SAS Institute Inc., Cary, NC) for windows was the statistical software that was used.

## Results

Table1 shows the nutritional composition of the flour samples as affected by soaking. The protein content of the 6 h-soaked*D. rotundata* and*D. alata* flour samples was lower than that of the control and this reduction was significant while the reduction in*D. alata* was insignificant. The protein content of 18 h-soaked*D. alata* flour sample was insignificantly different from the control. There was no significant difference in the moisture content of the 6- and 12 h-soaked*D. rotundata* flour samples but the moisture content of the 18 h-soaked*D. rotundata* flour sample was significant compared to the control. The highest moisture content (10.12%) was observed in 6 h-soaked*D. alata* flour sample while the 12 h-soaked*D. alata* had the lowest moisture content (8.16%). Table1 also reveals that there were no significant differences in the ash and fat contents of all the samples but their carbohydrate content ranged from 83.08% to 86.13%.

**Table 1:** Effect of soaking time on the proximate composition of yam flour in percentage.

Sample	Moisture %	Protein %	Fat %	CHO %	Fiber %	Ash %
*Dioscorea rotundata* 0 h	9.47 ± 0.12^b^	1.51 ± 0.01^b^	1.77 ± 0.20^a^	83.96 ± 0.78^cb^	1.93 ± 0.21^abc^	1.67 ± 1.15^a^
*Dioscorea alata* 0 h	9.97 ± 0.15^a^	0.91 ± 0.01^a^	1.89 ± 0.51^a^	83.61 ± 1.20^cb^	1.62 ± 0.39^abc^	2.00 ± 1.00^a^
*D. rotundata* 6 h	8.60 ± 0.78^c^	1.21 ± 0.26^a^	2.00 ± 0.34^a^	83.75 ± 1.13^cb^	2.14 ± 0.39^ab^	2.00 ± 0.00^a^
*D. alata* 6 h	10.12 ± 0.07^a^	0.88 ± 0.57^a^	2.00 ± 0.34^a^	83.08 ± 0.59^c^	2.26 ± 0.14^ab^	1.67 ± 0.58^a^
*D. rotundata* 12 h	8.41 ± 0.13^c^	1.16 ± 0.25^a^	1.11 ± 0.51^a^	85.97 ± 1.11^a^	1.35 ± 0.57^aa^	2.00 ± 0.00^a^
*D. alata* 12 h	8.16 ± 0.04^c^	1.10 ± 0.23^a^	1.44 ± 0.51^a^	86.13 ± 1.30^a^	1.17 ± 0.16^d^	2.00 ± 1.00^a^
*D. rotundata* 18 h	9.38 ± 0.05^b^	1.09 ± 0.01^a^	1.44 ± 0.51^a^	85.31 ± 0.74^ba^	1.43 ± 0.53^cd^	1.33 ± 0.58^a^
*D. alata* 18 h	9.30 ± 0.05^b^	0.94 ± 0.02^a^	2.22 ± 0.19^a^	83.18 ± 1.16^c^	2.36 ± 0.02^a^	2.00 ± 1.00^a^

Values are expressed as mean ± SD (*n* = 3); values within the same column followed by different superscripts are significantly different (*P* < 0.05).

*D. alata* which was used as the control had the highest dispersibility and its dispersibility was not significantly different from that of the 12 h-soaked*D. rotundata* flour sample as shown in Table2. The bulk density of the 18 h-soaked yam flour was significantly different from other samples with lesser soaking time.*D. rotundata* flour sample (18 h soaked) had the highest while 6 h-soaked*D. alata* flour sample had the lowest water absorption capacity and its value was not significantly different from*D. alata* that was used as control.

**Table 2:** Effect of soaking time on the functional properties of yam flour.

Samples	Dispersibility %	Bulk density g/mL	Water abs g/mL
*Dioscorea rotundata* 0 h	67.33 ± 0.58^b^	0.76 ± 0.02^a^	2.45 ± 0.01^g^
*Dioscorea alata* 0 h	68.83 ± 1.15^a^	0.75 ± 0.02^a^	2.50 ± 0.02^f^
*D. rotundata* 6 h	65.83 ± 0.29^c^	0.77 ± 0.01^a^	2.62 ± 0.01^e^
*D. alata* 6 h	67.67 ± 0.58^b^	0.78 ± 0.01^a^	2.45 ± 0.01^g^
*D. rotundata* 12 h	68.33 ± 0.58^ba^	0.74 ± 0.01^a^	2.86 ± 0.01^b^
*D. alata* 12 h	67.50 ± 0.50^b^	0.75 ± 0.01^a^	2.76 ± 0.03^c^
*D. rotundata* 18 h	64.83 ± 0.29^c^	0.66 ± 0.00^b^	2.91 ± 0.02^a^
*D. alata* 18 h	61.83 ± 0.29^d^	0.65 ± 0.02^b^	2.71 ± 0.03^d^

Values are expressed as mean ± SD (*n* = 3); values within the same column followed by different superscripts are significantly different (*P* < 0.05).

There was no difference (*P* > 0.05) in the color of all the yam flour samples at different soaking times as shown in Table3. Also the pH of the yam flour samples were different (*P* < 0.05) during the different soaking periods.

**Table 3:** Effect of soaking time on the pH and color of yam flour.

Sample	pH	Color
*Dioscorea rotundata* 0 h	6.40 ± 0.01^a^	1.81 ± 0.08^a^
*Dioscorea alata* 0 h	6.40 ± 0.01^a^	1.82 ± 0.07^a^
*D. rotundata* 6 h	5.82 ± 0.02^c^	1.82 ± 0.06^a^
*D. alata* 6 h	6.21 ± 0.01^b^	1.83 ± 0.06^a^
*D. rotundata* 12 h	5.54 ± 0.01^d^	1.81 ± 0.07^a^
*D. alata* 12 h	5.48 ± 0.02^e^	1.86 ± 0.03^a^
*D. rotundata* 18 h	5.10 ± 0.01^f^	1.85 ± 0.04^a^
*D. alata* 18 h	5.84 ± 0.02^c^	1.82 ± 0.08^a^

Values are expressed as mean ± SD (*n* = 3); values within the same column followed by different superscripts are significantly different (*P* < 0.05).

The lowest breakdown, final, and peak viscosity were observed in the 18 h-soaked*D. rotundata* flour sample (Table4). Pasting temperature ranged from 79.80 to 83.60°C. Flour samples made from*D. rotundata* soaked for 12 h had the highest peak viscosity followed by samples of*D. rotundata* soaked for 6 h and the control.*D. rotundata* soaked for 18 h flour sample had higher holding strength (471.50 RVU) compared to other samples The values of the breakdown for flour from*D. alata* variety samples were lower than that of the flour from*D. rotundata* cultivar except for*D. alata* soaked at 18 h. The final viscosities of the D.*rotundata* samples at different soaking times were not significantly different from each other. Setback values which are an index of retrogradation varied from 93.00 to 488.50 RVU.

**Table 4:** Effect of soaking time on the pasting properties of yam flour.

Sample	Peak viscosity (RVU)	Holding strength (RVU)	Breakdown (RVU)	Final viscosity (RVU)	Setback (RVU)	Peak time (Min)	Pasting temperature (°C)
*Dioscorea rotundata* 0 h	622.50 ± 487.20^a^	289.00 ± 752.36^c^	332.50 ± 265.17^a^	778.50 ± 785.60^ab^	488.50 ± 33.23^a^	4.70 ± 0.24^c^	79.80 ± 0.00^a^
*Dioscorea alata* 0 h	349.00 ± 46.67^d^	280.00 ± 49.50^c^	69.00 ± 2.83^f^	374.00 ± 9.90^d^	93.00 ± 59.40^d^	4.87 ± 0.09^bc^	81.93 ± 0.60^ab^
*D. rotundata* 6 h	594.501 ± 18.09^a^	405.00 ± 343.65a^b^	188.50 ± 225.57^b^	787.50 ± 137.89^ab^	381.50 ± 205.77^b^	5.03 ± 0.05^b^	80.68 ± 0.04 ^cd^
*D. alata* 6 h	454.25 ± 10.61^c^	334.00 ± 18.38^bc^	119.50 ± 7.78^d^	502.00 ± 7.07^c^	168.00 ± 11.31^bc^	4.83 ± 0.05^bc^	81.08 ± 0.67^bc^
*D. rotundata* 12 h	607.40 ± 364.87^a^	454.50 ± 550.84^a^	153.50 ± 185.97^c^	821.50 ± 245.37^a^	367.00 ± 305.47^b^	5.43 ± 0.14^a^	81.95 ± 0.57^ab^
*D. alata* 12 h	438.70 ± 26.16^c^	334.50 ± 30.41^bc^	104.00 ± 4.24^d^	493.00 ± 35.36^c^	159.50 ± 4.95^c^	5.43 ± 0.05^a^	82.35 ± 0.00^a^
*D. rotundata* 18 h	515.90 ± 9.90^b^	471.50 ± 33.23^a^	44.50 ± 43.13^ef^	723.00 ± 111.72^ab^	252.50 ± 144.96^b^	5.47 ± 0.09^a^	82.30 ± 0.00^a^
*D. alata* 18 h	439.90 ± 42.43^c^	352.00 ± 38.18^bc^	87.00 ± 4.24e	546.50 ± 51.62^a^	194.50 ± 13.44^c^	5.40 ± 0.00^a^	83.60 ± 0.49^b^

Values are expressed as mean ± SD (*n* = 3); values within the same column followed by different superscripts are significantly different (*P* < 0.05).

Table5 shows that there were significant differences (*P* ≤ 0.05) in the appearance, texture, taste, aroma, and the overall acceptability of the yam paste made from different flour samples. The color, taste, appearance, aroma, and the overall acceptability of the paste made from flour produced from the control sample of*D. rotundata* were significantly different from the appearance, color, taste, aroma, and the overall acceptability of paste made from 18 h-soaked*D. rotundata* flour. After the 18 h soaking period, the overall acceptability, taste, texture color, and appearance of*D. alata* were significantly different from*D. rotundata* at the same soaking time.

**Table 5:** Effect of soaking time on the sensory attributes of yam flour.

Sample	Appearance	Color	Texture	Taste	Aroma	Overall acceptability
*Dioscorea rotundata* 0 h	5.30 ± 2.60^c^	4.95 ± 2.24^c^	6.60 ± 1.93^d^	5.45 ± 2.28^abc^	4.65 ± 2.46^ab^	5.45 ± 2.61^c^
*Dioscorea alata* 0 h	5.90 ± 2.90^ab^	6.15 ± 2.37^cb^	3.40 ± 1.98^a^	4.40 ± 2.64^c^	4.10 ± 2.29^b^	5.30 ± 2.49^c^
*D. rotundata* 6 h	6.95 ± 1.43^bc^	6.50 ± 1.91^b^	6.95 ± 1.79^d^	6.25 ± 2.22^ba^	5.80 ± 2.26^a^	6.90 ± 1.92^ab^
*D. alata* 6 h	5.95 ± 2.16^cb^	6.00 ± 1.86^cb^	6.00 ± 2.20^cd^	5.70 ± 1.56^abc^	5.80 ± 2.04^a^	6.00 ± 1.84^abc^
*D. rotundata* 12 h	6.00 ± 1.52^cb^	6.10 ± 1.71^cb^	5.75 ± 2.36^cd^	5.35 ± 2.58^abc^	6.15 ± 2.13^a^	6.40 ± 1.79^abc^
*D. alata* 12 h	4.75 ± 2.24^c^	5.10 ± 2.15^c^	4.20 ± 2.38^ab^	4.35 ± 1.81^c^	4.90 ± 2.07^ab^	5.45 ± 1.64^c^
*D. rotundata* 18 h	7.55 ± 1.50^a^	7.85 ± 1.23^a^	6.85 ± 1.73^d^	6.45 ± 2.04^a^	6.15 ± 2.13^a^	7.20 ± 1.47^a^
*D. alata* 18 h	5.25 ± 2.05^c^	5.65 ± 1.50^cb^	4.90 ± 2.31^bc^	4.85 ± 2.37^bc^	5.75 ± 2.36^a^	5.60 ± 2.11^bc^

Values are expressed as mean ± SD (*n* = 3); values within the same column followed by different superscripts are significantly different (*P* < 0.05).

## Discussion

The variations in the protein content of the two yam varieties may be due to genetic composition of the varieties and environmental conditions (Woolfe 1987). The reduced protein content might be because of the progressive solubilization and movement of some nitrogenous substances into water used for soaking (Ukachukwu and Obioha 2000). All the samples had moisture content below 13% which is the standard for dry food samples as described by (Prinyawiwatkul et al. 1997), the result of the moisture content are similar to the previous reports of Jimoh and Olatidoye (2009). The insignificant differences obtained in the crude fat and ash values of the samples conform with the studies of Adejumo et al. (2013), who found that soaking had no significant effect on the fat and ash values of yam flour.

The dispersibility of a mixture in water indicates its ability to reconstitute, the higher the dispersibility of a mixture, the better is its reconstitution property (Ghavidel and Davood 2011), thus the result of this study shows that the 12 h-soaked*D. rotundata* flour sample will reconstitute better than others. The bulk density of the 18 h-soaked*D. rotundata* and*D. alata* flour samples was significantly different from other samples with lesser soaking time and this may be due to the starch particles becoming looser during this soaking period. It has been reported that bulk density reduces as soaking time increases.*D. rotundata* that was soaked for 18 h before being processed into flour had the highest water-holding capacity while*D. alata* soaked at 6 h had the lowest capacity, the water-holding capacity is also a function of the protein content of the yam flour (Kinsella 1976).

The insignificant difference observed in the color of all the yam flour samples might have been as a result of the uniformity in soaking temperature (50°C). Also the increase in soaking time might have caused a decrease in pH as a result of the actions of microorganisms which could have induced acidity.

The peak viscosity of all the*D. alata* samples were low compared to all the*D. rotundata* samples and this indicates that the carbohydrate components of*D. rotundata* flour samples will not breakdown easily and quickly like the*D. alata* samples until it is cooked properly. Peak viscosity has been reported to be an important parameter to processors so as to obtain a useable starch paste (Adeyemi 1989). The 18 h-soaked*D. rotundata* flour sample had the highest holding strength, holding strength indicates the capacity of a flour sample undergoing processing to resist shear stress and heating. The vulnerability of cooked starch granules to disintegrate into smaller components is measured as breakdown and this has been reported to affect the steadiness of flour products (Beta et al. 2001). Low breakdown value indicates that the stability of a flour sample is high under hot condition, therefore, the stability of 18 h-soaked*D. rotundata* flour sample will be higher under hot condition. Also, all the flour samples from*D. rotundata* can form thick and strong gel after cooking and cooling than samples from*D. alata* based on the observed insignificant differences in their final viscosity.

On the basis of the sensory analysis, the panelist preferred the taste, texture, color, and appearance of paste made from the 18 h-soaked*D. rotundata* flour to the paste made from other flour samples including the control sample. The soaking period might have influenced the color, texture, and appearance of*D. rotundata as* well as its acceptability. The result of some of its functional properties also showed that paste from 18 h soaked*D. rotundata* will have desirable sensory qualities.

## Conclusions

Soaking*D. rotundata* for 18 h before processing it into flour led to the retention of the nutrients of the yam flour except protein and improved the color, texture, and appearance of the paste that was developed from this flour. Also, a viscous and firm gel that will be stable at high temperature as well as a useable and acceptable starch paste with superior eating quality can be obtained by soaking*D. rotundata* for 18 h prior to processing.

## Conflict of Interest

None declared.
